# Supplementary material for: Effectiveness of art-based health education on anemia and health literacy among pregnant women in Western Nepal: A randomized controlled trial
Source: PLoS One. 2024 Sep 30;19(9):e0281789. doi: 10.1371/journal.pone.0281789 (PMC11441646; doi:10.1371/journal.pone.0281789)
Supplement: S1 File — (DOCX) [file pone.0281789.s002.docx]

**Title of the research**

Anemia in Nepali Pregnant Women: Risk Factors, Effectiveness of health education intervention for reducing anemia and improving health literacy in pregnant women of western Nepal

**Research Area**

Clinical research

**Summary of the proposal (Structured)**

This study aims to determine the independent effect of an face to face education program with art –based, illustrated booklet and nomogram without character.

**Background**

Anemia in pregnant women is a serious public health problem worldwide. Anemia during pregnancy is deﬁned as a hemoglobin (Hb) level below 11 g/dL by the World Health Organization (WHO). High prevalence of anemia has been related to high maternal deaths. Nepal is one of the countries with a high anemic pregnancy ratio and especially, 2.2% pregnant women suffer with severe anemia. Additionally, approximately half of all maternal deaths were related to postpartum hemorrhage with anemias.

**Rationale / Justification**

Education programs have been trialed as a way to improve anemia. However, such studies have only evaluated the education program using the materials, making it difﬁcult to assess the effect of the intervention of either the education program or distribution materials strategy. This study aims to determine the independent effect of an education program with booklet and nomogram without character.

**Objective**

**General objective**

1) To identify potential risk factors related to anemia (baseline survey), and

2) To determine the effectiveness of a health education program designed to improve anemia, with the aim to improve anemia in pregnant women in Nepal.

For number 1, we assess risks using logistic regression analysis with anemia and undernutrition as independent variables. For number 2, we use a randomized parallel-group comparison design to examine effectiveness by investigating hemoglobin levels, the incidence of anemia and health literacy in the following three groups: a) A group that underwent a health education program using an illustrated booklet and a nomogram, b) A group to which the illustrated booklet and nomogram are distributed, and c) A control group.

**Specific objectives**

To evaluate the effect of the education using art-based, booklet and nomogram for the target pregnant women including lower socioeconomic status, rural residence, illiteracy.

**Research Hypothesis**

1. This study expects that lower socioeconomic status, rural residence, unintended

pregnancy, illiteracy and working in agriculture are associated with teenage and anemic pregnancy.

1. Materials (booklet and nomogram) distribution only and Education with materials

have significantly higher hemoglobin changes than the control group.

3) Health literacy improves in the intervention group who received education.

**Study variables**

Maternal deaths are clustered around labor, delivery and the immediate postpartum period, with obstetric hemorrhage being the main medical cause of death.

This data shows that the extent of utilization of services pertaining to antenatal period is excellent for those other than illiterate women, low socio economical women. The roll of socio-economic factors in service utilization is clearly evident in study area. It clearly shows that as education level increases, the effect of health supports also increases. This intervention will indicate the effect of the education without character for illiterate and low socioeconomic pregnancy.

**Research Method**

A randomized, factorial design controlled trial will be conducted at the Western Regional Hospital, Nepal. Eligible pregnant women receiving prenatal care are randomized into 3 groups by block randomization with allocation concealment. Baseline hemoglobin at the recruitment phase and follow-up hemoglobin after delivery of recruitment are measured. Changes in hemoglobin levels and anemia prevalence are analyzed and compared between groups.

**Research design**

Purpose 1: Cross-sectional study design

Purpose 2: Randomized controlled trials between parallel groups design

**Description of research design**

Purpose 1: Baseline cross-sectional study to reveal potential risk factors related

to anemia

Purpose 2: Randomized controlled trials between parallel groups design to

compare three groups; 1) health education program group which receive

program using illustrated booklet and nomogram, 2) illustrated booklet and

nomogram distributed group which receive booklet and nomogram, and 3)

control group which receive normal prenatal checkup and does not receive any

intervention from the researcher, to validate the effectiveness.

**Study site and its Justification**

Data collection will be held at Western Regional Hospital, which offers pregnancy checkup in Pokhara, Nepal. This is the only public general hospital in Pokhara and owns 500 beds. It contains obstetrics, gynecology, pediatric with NICU, internal, surgical, emergency, psychiatry department and covers advanced perinatal medicine around Pokhara and its environs. For many pregnant women from mountain mountainous and rural areas in Kaski district visit this hospital, it is appropriate to implement this research, which examine risk factors for anemia and malnutrition such as socioeconomics, region, religion, race, and environment and evaluate a health education program for pregnant women with low self-care abilities and health literacy.

**Sampling unit**

Randomization with a block method is used to divide the enrolled women into 3 groups. The randomization is concealed using the numbered, sealed opaque envelope method overseen by an independent person. All eligible women according to the inclusion and exclusion criteria are approached consecutively. After they be provided written informed consent, baseline information on socio-demographic, nutrition, and obstetric characteristics is obtained using face-to-face interviews and from the medical records.

**Sample size**

**Baseline survey**

542 pregnant women is required.

Randomized controlled trial: RCT

138 pregnant women is required.

1. **Number of participants and Justification:**

**Baseline survey**

The sample size is estimated using the prevalence of anemia in pregnancy reported for the region (44.0%) （WHO 2015）. Using the Cochran formula, n = (Z^2^pq)/d^2^, where: n = sample size, Z = the z-score that corresponds with 95% confidence interval (1.96), P = proportion of anemia in pregnancy (44.0%, =0.44), q = proportion of antenatal attendants who are not anemic (1–0.44%, =0.56), d = margin of error set at 5% (0.05), a sample size of n =488. Assuming a 10% nonresponse rate a design effect of 2, a total sample size of 542 pregnant women is required.

Randomized controlled trial: RCT

The primary trial is a permuted block technique randomizes patients between groups within a set of study participants of pregnant women assigned to three groups: Control(n=46), Education program with materials and nomogram(n=46), Distribution materials and nomogram(n=46). The sample size was calculated Alpha was taken as 0.05 and beta to be 0.15. The power of the study was taken as 95%. Using mean±standard deviation scores of variable of hemoglobin from similar previous studies^38^, effect size 0.3571( Before and after difference 5g/SD14g Hb)showed a significant difference before and after the intervention in the intervention. Assuming a 10% dropout rate a design effect of 2, a total sample size of 138 pregnant women is required.

**Sampling Technique**

Randomization with a block method is used to divide the enrolled women into 3 groups. Research nurses then administer the surveys and interventions according to the randomization list. The research nurses who administer the surveys and interventions knew the group allocations but are blind to all participants and to the analysts of the survey results. All eligible women according to the inclusion and exclusion criteria are approached consecutively. After they be provided written informed consent, baseline information on socio-demographic, nutrition, and obstetric characteristics is obtained using face-to-face interviews and from the medical records.

**Criteria for sample selection**

Pregnant women who meet the following criteria will not be included in the study participants.

**Purpose 1:**

1) Pregnant women aged 16 years or older who were able to give informed consent,

2) Presence of a live single fetus in utero

3) Gestational age of 8–12 weeks at study entry

4) No underlying disease requiring regular oral medication

5) No cardiovascular disease, autoimmune disease, or any other condition affecting anemia

6) No condition that, in the opinion of the consenting health care professional, required exclusion from the study.

**Purpose 2:**

1) Pregnant women aged 16 years or older who were able to give informed consent,

2) Hb concentration of 7–10.9 g/dl

3) Presence of a live single fetus in utero

4) Gestational age of 8–12 weeks at study entry

5) No underlying disease requiring regular oral medication

6) No cardiovascular disease, autoimmune disease, or any other condition affecting anemia

7) No condition that, in the opinion of the consenting health care professional, required exclusion from the study.

**Data Collection Technique**

**Purpose 1:**

Questionnaires will be handed to the study participants after they be provided written informed consent. Research nurses read out the questionnaires in Nepalese, which are written in Nepalese, and fill-in their responses.

The level of hemoglobin will be measured in the laboratory of the research facility.

**Purpose 2:**

After the study participants provide written informed consent, Questionnaires will be handed to the study participants. Research nurses read out the questionnaires in Nepalese, which are written in Nepalese, and fill-in their responses

Depending on allocated group, the study participants will receive the following intervention.

1) Health education program group

The education group conduct three individual health education sessions during all gestational periods. Health education is provided by Nepalese research nurses trained by the principal investigator and co-investigators. The health education lasted approximately 10 minutes and is conducted face-to-face. Original text-free materials consisting of pictures, photographs, and nomograms are used in the health education sessions.

2) Illustrated booklet and nomogram distributed group

Pregnant women in the distribution group receive only the original educational materials and do not receive individualized health education.

3) Control group

Study participants will receive medical and nursing care from doctors and nurses of the hospital where they receive perinatal checkup, but will not receive any intervention from Research nurses. They receive the original educational materials in the third trimester (36–40 weeks) to ensure that they are not disadvantaged.

**Data collection tools**

1) Sociodemographic characteristics

Surname to confirm caste, age (birth date and year), height, weight, BMI, gestational age, residential area, religion, marital status, family structure, parity, number of children, birth interval, employment status (include participant’s husband and father’s data), household income, academic background (include husband’s data), literacy (include husband’s data), labor load (working time and burden), husband’s interest and understanding toward perinatal checkup, and health and nutrition (regular oral medication, smoking, alcohol, side stream smoke exposure, meal times, protein and vitamin intake, infection history of malaria, parasite, tuberculosis, HIV, AIDS within six months, iron supplement, intake of Nepal tea, and intake of vitamin C with iron supplement)

2) Level of hemoglobin and anemia incidence rate

3) Health literacy (using 14-item health literacy scale)

**Primary endpoint**

Hb levels are assessed again at 36–40 weeks’ gestational age as a follow-up study. The primary outcome measure is the change in Hb levels between baseline and follow-up studies in the three groups.

**Secondary endpoints** The HLS-14 developed by Suka et al. [1] is used as a measure of health literacy in Japanese patients. The HLS-14 is a comprehensive health literacy scale consisting of 14 items (5 functional literacy items on a 25-point scale, 5 communicative literacy items on a 25-point scale, and 4 critical literacy items on a 20-point scale), with a 70-point scale. Health literacy scores are assessed again at 36–40 weeks’ gestational age as a follow-up study. The primary outcome measure is the change in Health literacy level between baseline and follow-up studies in the three groups. HLS-14 Nepali version will be prepared in this study.

[１]Suka M, Odajima T, Kasai M, Igarashi A, Ishikawa H, Kusama M, et al. The 14-item health literacy scale for Japanese adults (HLS-14). Environ Health Prev Med. 2013;18: 407-415.

**Pretesting**

Previous literature (WHO2015) gives the estimate of anemic pregnancy in Nepal 44％ in population to be surveyed. Some intervention study indicated that the effect of education using educational materials. Although an education program would seem to be a simple and effective method to improve compliance and health outcomes such as nutritional status or reduction of blood pressure after interventions, there has been no study to test the effect of such an education program on Hb status and other birth outcomes during pregnancy. This study will be a pilot study of the intervention for anemic pregnancy.

**Validity and reliability of tool**

**1) Level of hemoglobin and anemia incidence rate**

Blood samples are collected by research nurses and analyzed at laboratories in cooperating research facilities for the assessment of blood hemoglobin levels. A hemoglobin cutoff value (WHO) of <11.0 g/dl is defined as anemia in pregnant women.

Pregnant women with anemia are screened by conducting a baseline study of pregnant women with pregnancies of gestational age 8–12 weeks.

**2) Health literacy**

The 14-item health literacy scale for Japanese adults (HLS-14) will be used to measure health literacy. The HLS-14 is a self-report measure which is consisted from 5 items on functional health literacy, 5 items on communicative health literacy, and 4 items on critical health literacy. Each scored 1-5 points, with a potential scale score of 14-70. Higher scores indicate greater health literacy. The reliability and validity among Japanese adults has been verified. Nepalese version will be developed through back translation.

**Potential Biases**

The permuted block technique randomizes patients between groups within a set of study participants, called a block. Treatment assignments within blocks are determined so that they are random in order but that the desired allocation proportions are achieved exactly within each block. In a 2-group trial with equal allocation and a block size of 6, 3 patients in each block would be assigned to the control and 3 to the treatment and the ordering of those 6 assignments would be random. For example, with treatment labels A and B, possible blocks might be: ABBABA, BABBAA, and AABABB. As each block is filled, the trial is guaranteed to have the desired allocation to each group. The main limitation of permuted block randomization is the potential for bias if treatment assignments become known or predictable.

Double-blinding may refer to blinding of both participants and health-care providers, investigators, data collectors, judicial assessors, or data analyzers. Although this study does not adopt double blind method, blinding of outcome assessors is possible and can theoretically be one of the most fundamental considerations. Additionally, dropout in longitudinal randomized controlled trials is a potential source of bias in terms of evidence based intervention. Bias in the estimate of treatment effect caused by dropout can also depend on the analytical approach used. We will omit missing data and analyze complete data only.

**Limitation of the study**

To enhance the generalizability and strengthen the evidence of effectiveness of education without character on anemia prevalence in pregnant women, a well-designed multicenter cluster randomized trial is needed.

We need to expand the study for large-scale investigation including several hospitals.

**Plan for supervision and monitoring**

The principal researcher will conduct training of the four Research nursess. The principal researcher and Research nursess will conduct the study together. When the principal researcher be absent, the collaborators will report on progress by e-mail or telephone to the principal researcher and discuss. Principal researcher will consult two supervisors, doctors from Western Regional Hospital, when needed.

1. **Plan for data management and analysis**

[Data management]

Data will be collected in a form making it hard to identify an individual. A secure USB drive and a computer detached from any network, which will be kept in Senior Consultant Surgeon Director’s room during data collecting period and principal researcher’ s after that period, will be used to store and analyze data. Questionnaires will also be strictly kept in a locked storage container in Senior Consultant Surgeon Director’s room during data collecting period and principal researcher’ s after that period. Storage periods will be 10 years from the end of the study or 10 years from the date of publication, whichever is the later. After the storage period, questionnaires will be shredded and an USB data erased irreversibly.

[Data analysis]

1: We assess risk using logistic regression analysis with anemia and undernutrition as independent factors. For confounding factors, we use a model that include age, parity, interval between births, body mass index (BMI), race, financial status, education, literacy, caste, workload, employment conditions, region, fruit/vegetable intake, smoking, exposure to second-hand smoke, and health literacy in order to determine the factors that are relate to anemia onset in pregnant women in Nepal.

2: One objective of this study is to determine the effectiveness of a health education program using a nomogram and illustrated booklet that are designed to improve anemia in pregnant women in Nepal. The study design is a randomized, parallel-group comparison. Effectiveness is asses by investigating a) A group that underwent a health education program using an illustrated booklet and a nomogram b) A group to which the illustrate booklet and nomogram are distribute, and c) A control group that receive only regular gynecological checkups without any intervention from researchers.

Descriptive statistics are used to analyze the data and calculate frequencies and percentages to describe the characteristics of the study participants. Continuous variables are evaluated using the mean and standard deviation (SD). Fisher’s exact test and the Chi-square test are used to analyze association of categorical variables between the three groups. To evaluate the primary outcome (Hb levels), These are analyzed using analysis of variance (one-way analysis of variance or the Kruskal-Wallis test) and multiple comparison (the Tukey test or the Mann-Whitney U test). To assess health literacy, the Wilcoxon signed rank sum test is used to calculate the change in total health literacy scale scores and subscale scores before and after the intervention.

**Expected outcome of the research results**

Purpose 1: This research will lead to clarify risk factors complicatedly related to anemia in Nepali pregnant women

Purpose 2: This research corrects the impacts of factors relating to anemia in Nepali pregnant women and leads to investigate effective health education program to improve anemia.

1. **Plan for utilization of research findings**

Research findings will be submitted to academic journals and shared with women in Nepal, especially pregnant women and health care providers who support them.
