# Supplementary material for: Effectiveness of art-based health education on anemia and health literacy among pregnant women in Western Nepal: A randomized controlled trial
Source: PLoS One. 2024 Sep 30;19(9):e0281789. doi: 10.1371/journal.pone.0281789 (PMC11441646; doi:10.1371/journal.pone.0281789)
Supplement: Attachment — Submitted filename: PONE-D-22-35514_reviewer.pdf [file pone.0281789.s004.pdf]

PLOS ONE

# Effectiveness of art-based health education on anemia and health literacy among pregnant women in Western Nepal: A randomized controlled trial

--Manuscript Draft--

| | |
|---|---|
| Manuscript Number: | PONE-D-22-35514 |
| Article Type: | Clinical Trial |
| Full Title: | Effectiveness of art-based health education on anemia and health literacy among pregnant women in Western Nepal: A randomized controlled trial |
| Short Title: | Effectiveness of art-based health education on anemia and health literacy among pregnant women |
| Corresponding Author: | Hiroko Sakai<br>Kansai Medical University: Kansai Ika Daigaku<br>Osaka, JAPAN |
| Keywords: | |
| Abstract: | Objective: Anemia among Nepalese pregnant women has not decreased in the past 20 years despite various interventions. Because Nepalese pregnant women vary widely in literacy levels and cultural backgrounds and are reluctant to make decisions about their health, general interventions are insufficient to improve maternal anemia. This study aimed to assess the effectiveness of "face-to-face health education using educational material created using pictures, photos, and nomograms" in reducing anemia and improving health literacy.<br>Methods: A total of 156 Nepalese pregnant women with hemoglobin (Hb) levels below 11.0 g/dl were divided into three groups: the education group, received three sessions of face-to-face health education using art-based material unaffected by literacy skills; the distribution group, received material used in the education group; and the control group, received general perinatal checkups. Hb levels and health literacy scores were assessed at baseline early pregnancy (8–12 weeks) and late pregnancy (36–40 weeks). A Nepalese version of the 14-item Health Literacy Scale (HLS-14) was developed to assess health literacy.<br>Results: Hb levels (mean±SD) before and after the intervention were compared among the three groups. The changes in Hb levels from before to after the intervention were 1.40 ± 0.84 g/dl, 0.96 ± 1.21 g/dl in the group, and 0.87 ± 1.11 g/dl in the education, distribution, and control groups, respectively, with a significantly higher percentage change in the education group (P < 0.035). Comparing the change in health literacy scale (total score, number of subscales) scores before and after the intervention, there was a significant difference in the total score in the education group (P < 0.001). Significant differences in functional literacy (P<0.012), communication literacy (P<0.004), and critical literacy (P<0.014) were found only in the education group.<br>Conclusion: Continuous face-to-face health education using literacy material significantly reduced anemia and improved health literacy among Nepalese pregnant women. |
| Order of Authors: | Hiroko Sakai |
| | Rina Kawata |
| | Rajesh Adhikari |
| | Yoko Oda Thapa |
| | Tulsi Ram Bhandari |
| Additional Information: | |
| Question | Response |
| **Financial Disclosure**<br><br>Enter a financial disclosure statement that describes the sources of funding for the | •Initials of the authors who received each award: H S<br>•Grant numbers awarded to each author: Hiroko Sakai JSPS KAKENHI Grant Numbers 18k17592,22k0026.<br>•The full name of each funder: Grant-in-Aid for Young Scientists, Fostering Joint |

*Powered by Editorial Manager® and ProduXion Manager® from Aries Systems Corporation*

| | |
|---|---|
| work included in this submission. Review the submission guidelines for detailed requirements. View published research articles from *PLOS ONE* for specific examples.<br><br>This statement is required for submission and **will appear in the published article** if the submission is accepted. Please make sure it is accurate.<br><br>**Unfunded studies**<br>Enter: *The author(s) received no specific funding for this work.*<br><br>**Funded studies**<br>Enter a statement with the following details:<br>• Initials of the authors who received each award<br>• Grant numbers awarded to each author<br>• The full name of each funder<br>• URL of each funder website<br>• Did the sponsors or funders play any role in the study design, data collection and analysis, decision to publish, or preparation of the manuscript?<br>• **NO** - Include this sentence at the end of your statement: *The funders had no role in study design, data collection and analysis, decision to publish, or preparation of the manuscript.*<br>• **YES** - Specify the role(s) played.<br><br>* typeset | International Research (B)<br>•URL of each funder website: Japan Society for the Promotion of Science (jsps.go.jp)<br><br>The funders had no role in study design, data collection and analysis, decision to publish, or preparation of the manuscript./ |
| **Competing Interests**<br><br>Use the instructions below to enter a competing interest statement for this submission. On behalf of all authors, disclose any competing interests that could be perceived to bias this work—acknowledging all financial support and any other relevant financial or non-financial competing interests.<br><br>This statement is required for submission and **will appear in the published article** if the submission is accepted. Please make sure it is accurate and that any funding sources listed in your Funding Information | The authors have declared that no competing interests exist. |

*Powered by Editorial Manager® and ProduXion Manager® from Aries Systems Corporation*

| | |
|---|---|
| later in the submission form are also declared in your Financial Disclosure statement.<br><br>View published research articles from *PLOS ONE* for specific examples.<br><br>**NO authors have competing interests**<br><br>Enter: *The authors have declared that no competing interests exist.*<br><br>**Authors with competing interests**<br><br>Enter competing interest details beginning with this statement:<br><br>*I have read the journal's policy and the authors of this manuscript have the following competing interests: [insert competing interests here]*<br><br>* typeset | |
| **Ethics Statement**<br><br>Enter an ethics statement for this submission. This statement is required if the study involved:<br><br>• Human participants<br>• Human specimens or tissue<br>• Vertebrate animals or cephalopods<br>• Vertebrate embryos or tissues<br>• Field research<br><br>Write "N/A" if the submission does not require an ethics statement.<br><br>General guidance is provided below. Consult the submission guidelines for detailed instructions. **Make sure that all information entered here is included in the Methods section of the manuscript.** | The survey was conducted with the approval of the Research Ethics Committee (No. 2018180) of the Kansai Medical University to which the principal investigator belongs, as well as the Institutional Review Committees of the Nepal Health Research Council (No. 358) and Western Regional Hospital (No. 64). Clinical trial enrollment for this study was not completed prior to the start of the study, as this was an interventional study, and due to a lack of understanding regarding the need for clinical trial enrollment. An analysis plan was presented in the study protocol and the analysis was conducted according to the analysis plan. The authors confirm that all ongoing and related trials for this drug/intervention are registered. The study participants were pregnant women who understood the study and gave written consent before enrollment. |

Powered by Editorial Manager® and ProduXion Manager® from Aries Systems Corporation

| | |
|---|---|
| **Format for specific study types**<br><br>**Human Subject Research (involving human participants and/or tissue)**<br>• Give the name of the institutional review board or ethics committee that approved the study<br>• Include the approval number and/or a statement indicating approval of this research<br>• Indicate the form of consent obtained (written/oral) or the reason that consent was not obtained (e.g. the data were analyzed anonymously)<br><br>**Animal Research (involving vertebrate animals, embryos or tissues)**<br>• Provide the name of the Institutional Animal Care and Use Committee (IACUC) or other relevant ethics board that reviewed the study protocol, and indicate whether they approved this research or granted a formal waiver of ethical approval<br>• Include an approval number if one was obtained<br>• If the study involved *non-human primates*, add *additional details* about animal welfare and steps taken to ameliorate suffering<br>• If anesthesia, euthanasia, or any kind of animal sacrifice is part of the study, include briefly which substances and/or methods were applied<br><br>**Field Research**<br><br>Include the following details if this study involves the collection of plant, animal, or other materials from a natural setting:<br>• Field permit number<br>• Name of the institution or relevant body that granted permission | |
| **Data Availability**<br><br>Authors are required to make all data underlying the findings described fully available, without restriction, and from the time of publication. PLOS allows rare exceptions to address legal and ethical concerns. See the PLOS Data Policy and FAQ for detailed information. | No - some restrictions will apply |

| | |
|---|---|
| A Data Availability Statement describing where the data can be found is required at submission. Your answers to this question constitute the Data Availability Statement and **will be published in the article**, if accepted.<br><br>**Important:** Stating 'data available on request from the author' is not sufficient. If your data are only available upon request, select 'No' for the first question and explain your exceptional situation in the text box.<br><br>Do the authors confirm that all data underlying the findings described in their manuscript are fully available without restriction? | |
| **Describe where the data may be found in full sentences. If you are copying our sample text, replace any instances of XXX with the appropriate details.**<br><br>• If the data are **held or will be held in a public repository**, include URLs, accession numbers or DOIs. If this information will only be available after acceptance, indicate this by ticking the box below. For example: *All XXX files are available from the XXX database (accession number(s) XXX, XXX.).*<br>• If the data are all contained **within the manuscript and/or Supporting Information files**, enter the following: *All relevant data are within the manuscript and its Supporting Information files.*<br>• If neither of these applies but you are able to provide **details of access elsewhere**, with or without limitations, please do so. For example:<br><br>*Data cannot be shared publicly because of [XXX]. Data are available from the XXX Institutional Data Access / Ethics Committee (contact via XXX) for researchers who meet the criteria for access to confidential data.*<br><br>*The data underlying the results presented in the study are available from (include the name of the third party* | Data cannot be released to the public because it contains data requiring ethical considerations. Researchers who meet the criteria for access to confidential data may obtain the data from the Corresponding Author. |

| | |
|---|---|
| *and contact information or URL).*<br>• This text is appropriate if the data are owned by a third party and authors do not have permission to share the data.<br><br>* typeset | |
| Additional data availability information: | |

Manuscript Click here to access/download;Manuscript;Manuscript.docx



# Effectiveness of art-based health education on anemia and health literacy among pregnant women in Western Nepal: A randomized controlled trial

Short title: Effectiveness of art-based health education on anemia and health literacy among pregnant women

Hiroko Sakai[1*], Rina Kawata[2],Rajesh Adhikari[3],Yoko Oda Thapa[4], Tulsi Ram Bhandari[5]

[1]Kansai Medical University Faculty of Nursing Graduate School of Nursing, Hirakata, Japan
[2]Division of Health Science, Graduate School of Medicine, Suita, Osaka University, Osaka, Japan
[3] Gynaecology Department of Pokhara Academy of health sciences, Pokhara, Nepal.
[4]Action Research for Community Health International, Damauli, Nepal
[5] School of Health and Allied Sciences, Faculty of Health Sciences, Pokhara University, Pokhara, Nepal

*Corresponding Author

E-mail: sakaihir@hirakata.kmu.ac.jp (HS)

**Trial Registration:** UMIN Clinical Trials Registry (UMIN-CTR), URL: https://www.umin.ac.jp/ctr/ and the registration number - UMIN000049603

# Abstract

**Objective:** Anemia among Nepalese pregnant women has not decreased in the past 20 years despite various interventions. Because Nepalese pregnant women vary widely in literacy levels and cultural backgrounds and are reluctant to make decisions about their health, general interventions are insufficient to improve maternal anemia. This study aimed to assess the effectiveness of "face-to-face health education using educational material created using pictures, photos, and nomograms" in reducing anemia and improving health literacy.

**Methods:** A total of 156 Nepalese pregnant women with hemoglobin (Hb) levels below 11.0 g/dl were divided into three groups: the education group, received three sessions of face-to-face health education using art-based material unaffected by literacy skills; the distribution group, received material used in the education group; and the control group, received general perinatal checkups. Hb levels and health literacy scores were assessed at baseline early pregnancy (8–12 weeks) and late pregnancy (36–40 weeks). A Nepalese version of the 14-item Health Literacy Scale (HLS-14) was developed to assess health literacy.

**Results:** Hb levels (mean±SD) before and after the intervention were compared among the three groups. The changes in Hb levels from before to after the intervention were 1.40 ± 0.84 g/dl, 0.96 ± 1.21 g/dl in the group, and 0.87 ± 1.11 g/dl in the education, distribution, and control groups, respectively, with a significantly higher percentage change in the education group ($P < 0.035$).

Comparing the change in health literacy scale (total score, number of subscales) scores before and after the intervention, there was a significant difference in the total score in the education group ($P < 0.001$). Significant differences in functional literacy ($P<0.012$), communication literacy ($P<0.004$), and critical literacy ($P<0.014$) were found only in the education group.

**Conclusion:** Continuous face-to-face health education using literacy material significantly reduced anemia and improved health literacy among Nepalese pregnant women.

**Trial Registration number**: UMIN000049603

# Introduction

Health literacy (HL) has been defined as “the cognitive and social skills which determine the motivation and ability of individuals to gain access to, understand, and use information in ways that promote and maintain good health” [1]. In developing countries, many social determinants of health affect people’s lives, including poverty, gender inequality, educational disparities, exploitation, violence, and injustice. These factors contribute to illness and death among the poor and marginalized [2]. In the Federal Democratic Republic of Nepal (hereinafter referred to as Nepal), there are significant health disparities due to social determinants of health, and healthcare systems and support for health maintenance and promotion are still inadequate.

In particular, Nepalese women have difficulty making decisions and taking health-related actions of their own volition due to various social background-related factors, including religion, tradition, gender roles, and caste; thus, they need to be empowered [3]. Health literacy is a personal skill that can be developed and promotes independence in health care based on the patient’s own decisions [4]. The potential of health literacy to reduce inequalities, increase health system responsiveness, and promote the achievement of the United Nations Sustainable Development Goals is gaining attention [5].

There is growing awareness worldwide of the ethical imperative for patients to participate in decision-making regarding the health care they receive. As patient decision-making has been shown

to be effective in behavior change and health outcomes, there has also been recognition of the importance of improving patient HL and supporting patient self-determination [6,7]. These effective health behaviors and health outcomes include reduced patient anxiety and increased patient knowledge, patient satisfaction with treatment decisions, and treatment adherence [8].

While substantial progress has been made in many aspects of healthcare delivery in Nepal, perinatal mortality rates remain high [9]. Malnutrition in pregnant women has a variety of negative effects on mother and child.

Anemia during pregnancy is associated with an increased risk of perinatal and maternal death, preterm delivery, and low birth weight [10–13]. The lack of improvement in perinatal mortality is related to the nutritional status of pregnant women: 46% of pregnant women in Nepal have a body mass index (BMI) below 18.5, indicating a high rate of undernutrition [14]. In addition, 40% of pregnant women in Nepal are anemic according to the latest data, with no significant improvement in the past 20 years [15].

Most of the previous studies on anemia and malnutrition among Nepalese pregnant women are cross-sectional studies. In addition, iron supplements have been distributed to all pregnant women as a national policy, but the anemia status of pregnant women before and after the intervention has not been assessed. Furthermore, there has been no evaluation of iron supplementation compared to a control group, and an association between low compliance to iron supplementation and high anemia

rates among Nepalese pregnant women has been noted [16].

In view of the current lack of improvement in maternal hemoglobin levels in Nepal, the distribution of iron supplements alone is not sufficient for reducing anemia [17]. In particular, the causes of health disparities among Nepalese women are diverse, pointing to the need for long-term observation aimed at improving health literacy in addition to health outcomes when interventions for health promotion are implemented [18].

Health literacy interventions consist of lectures, passive teaching, one-way delivery of information, distribution of brochures and leaflets, and health education sessions using visual aids [19]. Traditional methods alone are inadequate for pregnant women in Nepal, who have varying literacy levels and cultural backgrounds and are reluctant to make decisions about their own health. Health education for persons with low health literacy requires the use of material that takes into account the comprehension skills of the target population, and material that includes pictures and diagrams is significantly understood [20]. Therefore, this study aimed to evaluate the effectiveness of "face-to-face health education using educational material created using pictures, photos, and nomograms without text" to improve hemoglobin in Nepalese pregnant women. Furthermore, the study sought to assess whether the intervention improved the health literacy of Nepalese pregnant women.

# Materials and methods

# Ethics Statement

The survey was conducted with the approval of the Research Ethics Committee (No. 2018180) of the Kansai Medical University to which the principal investigator belongs, as well as the Institutional Review Committees of the Nepal Health Research Council (No. 358) and Western Regional Hospital (No. 64). Clinical trial enrollment for this study was not completed prior to the start of the study, as this was an interventional study, and due to a lack of understanding regarding the need for clinical trial enrollment. An analysis plan was presented in the study protocol and the analysis was conducted according to the analysis plan. The authors confirm that all ongoing and related trials for this drug/intervention are registered. The study participants were pregnant women who understood the study and gave written consent before enrollment. Nepalese research nurses, who had received prior training and understood the purpose and content of the study, administered the survey using a Nepali questionnaire with an ID number. The questions and answer choices on the questionnaire were explained orally (Nepali) to the participants by the research nurse, and the oral (Nepali) responses were transcribed onto the survey form. If the participants were aged <16 years, consent was obtained from both the participants and their guardians, and informed consent was obtained for those participants for whom informed consent would be difficult, with explanations that could be understood.

## Study design

This study employed a randomized controlled trial (RCT) design and adheres to the Consolidated Standards of Reporting Trials (CONSORT) Statement. The study had a randomized parallel-group comparison design in which a single-blind test was used to compare [1] an education group using original educational material [2], a distribution group receiving only educational material, and [3] a control group receiving no intervention from the researchers and only a routine antenatal checkup.

## Eligibility criteria

Anemia tests were performed on 609 pregnant women with pregnancies of gestational age 8–12 weeks who consented to the study, and 201 anemic pregnant women with Hb levels of <11.0 g/dl were screened. Inclusion criteria for screening and consent were as follows: (1) pregnant women aged 16 years or older who were able to give informed consent, (2) an Hb concentration of 7–10.9 g/dl, (3) presence of a live single fetus in utero, (4) gestational age of 8–12 weeks at study entry, (5) no underlying disease requiring regular oral medication, (6) no cardiovascular disease, autoimmune disease, or any other condition affecting anemia, and (7) no condition that, in the opinion of the consenting health care professional, required exclusion from the study.

An obstetrician, a co-investigator at the collaborating institution, verified the eligibility of the participants. Five research nurses explained the study to women diagnosed with gestational anemia

and received a signed research consent form.

## Randomization and allocation

The participants were assigned to one of three groups by block randomization. They were assigned to the three groups according to a six-block, computer-generated randomization order created by a statistician not involved in the study. Research nurses then administered the surveys and interventions according to the randomization list. The five research nurses who administered the surveys and interventions knew the group allocations but were blinded to all participants and to the analysts of the survey results. The results were analyzed using Microsoft Excel (Microsoft Corp., Redmond, WA, USA) (Fig 1).

**Fig 1. Flowchart of study participation and follow-up at Western Regional Hospital**

## Study area and participants

Participants were recruited from pregnant Nepalese women who came to Western Regional Hospital for prenatal care. Western Regional Hospital is the only public general hospital in Pokhara, the second largest city in Nepal, located approximately 200 km west of the capital Kathmandu. Furthermore, it has the highest number of deliveries among the facilities in Pokhara. Pregnant

women from surrounding areas (urban, mountain, and rural) use this hospital, and it was chosen as a suitable location to study pregnant women with social determinants of health that contribute to health disparities. The date range for participant recruitment and follow-up survey was from March 2019 to March 2020. The authors confirm that all ongoing and related trials for this drug/intervention are registered

## Baseline and follow-up surveys

All participants completed a baseline survey (questionnaire including Hb levels, socioeconomic status, health status, health literacy scale, and height and weight measurements) at the first parental checkup. A follow-up survey (including Hb level, confirmation of iron medication, and health literacy scale) was conducted at 36–40 weeks of gestation. Data were collected by research nurses through face-to-face interviews using the questionnaire. Five research nurses who had received prior training from the principal investigator and co-investigators on how to conduct interviews using the questionnaire demonstrated the survey. Hemoglobin measurements were performed by laboratory technicians at collaborating facilities using the same equipment and analytical methods.

## Intervention

The education group underwent three individual health education sessions during all gestational

periods. Health education was provided by Nepalese research nurses trained by the principal investigator and co-investigators. The health education lasted approximately 10 minutes and was conducted face-to-face. Original text-free material consisting of pictures, photographs, and nomograms were used in the health education sessions. Pregnant women in the distribution group received only the original educational material and did not receive individualized health education. Pregnant women in the control group received a general prenatal health examination. They received the original educational material in the third trimester (36–40 weeks) to ensure that they were not disadvantaged.

We also made a teaching manual for the research nurses who conducted the education sessions. The manual described perinatology, nutrition, lifestyle, and recipes, and the opinions of two Nepalese obstetricians were incorporated to determine whether the text matched the description of the original educational material and whether the content was appropriate for education targeting Nepalese pregnant women.

The face-to-face intervention corresponded to the level of understanding, amount of knowledge, and health concerns of pregnant women, and included the following items: medical knowledge about anemia among pregnant women, the effects of anemia on mother and child, the importance of iron supplementation, Nepali women's food culture and nutritional imbalances, junk food consumption, iron-rich food consumption and cooking methods to prevent and improve anemia, and precautions

and side effects when taking iron pills and how to deal with them. As part of the nutrition education program, we used illustrations and photos to introduce menus and recipes that improve anemia using inexpensive and locally available food items.

## Data collection

### Baseline study

Information on social background relating to health determinants was obtained in a baseline study. Questions included family name, to identify ethnicity and caste; age (date of birth); gestational age; place of residence; religion; age at marriage; family structure; pregnancy history; number of children; birth interval; employment of mother and husband; occupation of mother, husband, and father; household income; education of mother and husband; and literacy of mother and husband. Questions on health status included underlying medical conditions and pregnancy complications. Questions regarding daily life included burden (working hours and sense of burden); husband’s interest in and understanding of prenatal care; smoking and drinking; exposure to secondhand smoke; frequency of meals; frequency of meat, fish, and legume consumption; age; treatment history of various infectious diseases, including HIV and AIDS; and iron supplementation. The questionnaire was developed by the principal investigator with reference to previous studies and translated into Nepali.

### Intervention Follow-up study

A follow-up study was conducted in all groups. Questionnaires were administered during prenatal checkups at a gestational age of 36–40 weeks. The questionnaire consisted of the 14-item Health Literacy Scale (HLS-14) and iron medication status. Hemoglobin levels were also tested.

## Primary endpoint

### Hemoglobin level (g/dl)

Blood samples were collected by research nurses and analyzed at laboratories in cooperating research facilities for the assessment of blood hemoglobin levels. A hemoglobin cutoff value (WHO) of <11.0 g/dl was defined as anemia in pregnant women. Pregnant women with anemia were screened by conducting a baseline study of pregnant women with pregnancies of gestational age 8–12 weeks. Women diagnosed as anemic during this screening were recruited for the intervention study.

Hb levels were assessed again at 36–40 weeks of gestational age as a follow-up study. The primary outcome measure was the change in Hb levels between baseline and follow-up studies in the three groups.

## Secondary endpoints

## Health literacy

The HLS-14 developed by Suka et al. [21] was used as a measure of health literacy in Japanese patients. The HLS-14 is a comprehensive health literacy scale consisting of 14 items (5 functional literacy items on a 25-point scale, 5 communicative literacy items on a 25-point scale, and 4 critical literacy items on a 20-point scale), with a 70-point scale. Functional literacy refers to the basic skills of reading and writing to function effectively in daily life. Communicative literacy refers to more advanced skills for actively participating in daily life, obtaining information, understanding health status from various forms of communication, and applying new information to changing health conditions. Critical literacy refers to more advanced skills in critically analyzing information and using that information to better health conditions.

The HLS-14 scale has demonstrated reliability and validity primarily with Japanese adults and has been validated in other countries [22].

# Development of a Nepalese version of the HLS-14

Approval for the development of the Nepalese version of the HLS-14 was obtained from the corresponding author in Japan, the developer of the HLS-14 [21]. The equivalence of interpretations of the meaning of each item was discussed among the two Nepalese research collaborators, the Japanese principal investigator, and a Japanese researcher living in Nepal. They discussed the

constructs of the original HLS-14 and their appropriateness for application among Nepalese people. Discussions were conducted in English and Nepali.

To ensure equivalence of meaning and interpretation, the original questionnaire was translated directly from Japanese into Nepali. This was done by one Nepali with a master’s degree in economics, who is a native speaker of Nepali and fluent in English and Japanese, and one Japanese national with a master’s degree in public health living in Nepal. Translations were made from the Nepali translation into English and from English into Japanese by translators (one native Nepali speaker fluent in English and one native Japanese speaker fluent in English) who were unaware of the purpose of this study.

It was then confirmed that the four researchers had a common understanding of each item and the words in each item, and that equivalence of meanings and items could be guaranteed. As a result, one word was modified for one item to make it more understandable. After the abovementioned process, the Nepali version of the HLS-14 questionnaire was completed.

Five Nepalese research nurses who understood the main purpose of this study were asked to check the Nepali version of the HLS-14 for any inconsistencies or discrepancies in meaning or interpretation.

## Body mass index

Height and weight measurements were taken during the first prenatal checkup (baseline study). The seca-213 portable height meter was used for height measurement and the Tanita BC-314

(Tanita Corp., Tokyo, Japan) body composition scale was used for weight measurement. Weights were compared with three other scales to ensure that there were no errors. BMI was calculated as weight (kg)/square of height ($m^2$). WHO cutoff values for BMI were used: underweight, <18.5; normal weight, 18.5–24.9; and overweight/obese, >25.0.

## Supplementation of Hemferon-S tablets

All participants in the intervention and control arms received one tablet of Fefo (200 mg of dried Ferrous Sulphate + 0.40 mg Folic Acid) supplement once daily. The Government of Nepal launched the Iron Intensification Program (IIP) in 2003 [23], under which iron and folic acid (IFA) are distributed to all pregnant women. The pregnant women are provided supplements from the first perinatal checkup until 45 days postpartum.

## Sample size

In a previous study, 320 pregnant Nepalese women were randomly divided into four groups (education group, iron count group, education + iron count group, and control group), and the effects of health education were examined in terms of hemoglobin levels and anemia incidence [13]. To demonstrate the effect of health education using pictures and graphs, we estimated and calculated an effect size of 0.3571, α=0.05, power=0.95, and a dropout rate of 10%, with a sample size of 138 cases

and 46 cases each in the three groups (Gpower v.3.1).

## Analysis method

Using confirmatory factor analysis, fit indices (comparative fit index [CFI], Tucker-Lewis index [TLI], and root mean square error of approximation [RMSEA]) were calculated for 609 pregnant women to confirm the factor structure of the model of the Nepali version of the HLS scale and validate the goodness of fit. Furthermore, Cronbach's Alpha coefficient was used to evaluate the reliability of the scale.

Descriptive statistics were used to analyze the data and calculate frequencies and percentages to describe the characteristics of the study participants. Continuous variables were evaluated using the mean and standard deviation (SD). Fisher's exact test and the Chi-square test were used to analyze the associations of categorical variables between the three groups. To evaluate the primary outcome (Hb levels), analysis of variance (ANOVA) was used to compare Hb levels before and after the intervention and the rate of change before and after the intervention among the three groups.

To assess health literacy (secondary outcome), the Wilcoxon signed rank sum test was used to calculate the change in total health literacy scale scores and subscale scores before and after the intervention. All probability values less than 0.05 were considered statistically significant. Statistical analysts analyzed data blinded to grouping. All outcomes were analyzed using all available data in

an intention-to-treat framework. The data were statistically analyzed using IBM SPSS Statistics, version 28.0 (IBM Japan, Tokyo, Japan).

# Results

Out of the 609 pregnant women with pregnancies of gestational age 8–12 weeks who underwent prenatal health examinations during the study period and who consented to the study for anemia screening, 201 were diagnosed as anemic pregnant women. The following patients were excluded: 16 women who did not consent to the study, 1 woman with heart disease, 2 women with twins, 1 woman with severe anemia, and 25 pregnant women over 13 weeks of gestation. A total of 156 participants participated in the study and were randomly assigned to one of three groups. Eighteen participants had withdrawn from the study by follow-up for miscarriage, premature birth, stillbirth, or transfer to another hospital, leaving a total of 138 participants in the final analysis: 49 in the education group, 44 in the distribution group, and 45 in the control group (Table 1). The participants had a mean age of 24.4±4.5 years, mean age at marriage of 20.2±3.2 years, 23 (16.7%) were in their teens, and 72 (52.2%) in their 20s at marriage. One hundred and twelve (81.2%) participants were urban dwellers, 26 (18.8%) were rural dwellers, 127 (92.0%) were Hindu, 68 (49.3%) were extended family members, and 70 (50.7%) were nuclear family members. By ethnicity and caste, 65 (47.1%) were Brahman Chettri, a high caste, and 40 (29.0%) were Janajati, a collective term for minorities,

such as Gurung Magar, Newar/Sheresrha, and Thakali who do not belong to Hilly Hindu, to which upper caste and lower caste belong. Of the respondents, 88.4% had secondary education or higher. The literacy rate was 97.8%, and 96.4% of the spouses were literate. None of the pregnant women were current smokers or drinkers, and 122 (88.4%) reported taking iron tablets regularly. Fifty-eight (42.0%) had a spouse who was away from home as a migrant/short-term worker (Table 2).

**Table 1. Characteristics of the participants ([illegible]38)**

| | | |
|---|---|---|
| **Age (years)** | Mean±SD ([illegible]ge) | 24.4 ± 4.5 (16–38) |
| **Age of marriage (years)** | Mean±SD (range) | 20.0±3.4 (14–31) |
| **Age of first childbirth (years)** | Mean±SD (range) | 21.1±3.2 (15–32) |
| | | n (%) |
| **Residence, n (%)** | | |
| | Urban | 112 (81.2) |
| | Rural | 26 (18.8) |
| **Religion, n (%)** | | |
| | Hindus | 127 (92.0) |
| | Buddhist | 6 (4.3) |
| | Muslim | 0 (0.0) |
| | Others | 5 (3.6) |
| **Type of family, n (%)** | | |
| | Extended family | 68 (49.3) |
| | Immediate family | 70 (50.7) |
| **Ethnicity, caste, n (%)** | | |
| | Bramin, Chettri | 65 (47.1) |
| | Janajati | 40 (29.0) |
| | Dalit | 29 (21.0) |
| | Muslim, Madeshi, Others | 4 (2.9) |
| **Pregnancy history, n (%)** | | |
| | 1 | 70 (50.7) |
| | 2 | 45 (32.6) |

| | | |
|---|---|---|
| | 3 or more | 23 (16.7) |
| **Number of children, n (%)** | | |
| | 0 | 77 (55.8) |
| | 1 | 53 (38.4) |
| | 2 | 8 (5.8) |
| **Duration from last delivery, n (%)** | | |
| | First pregnancy | 77 (55.8) |
| | <1 year | 1 (0.7) |
| | 1–2 years | 25 (18.1) |
| | 3 or more years | 35(25.4) |
| **Treatment history, n (%)** | | |
| | None | 137 (99.3) |
| | Worms (parasite infection) | 1 (0.7) |
| **Smoking history, n (%)** | | |
| | No, never | 137 (99.3) |
| | Stopped during pregnancy | 1 (0.7) |
| **Family member's smoking history, n (%)** | | |
| | Yes | 34 (24.6) |
| | No | 104 (75.4) |
| **History of alcohol drinking, n (%)** | | |
| | Yes | 0 (0.0) |
| | No | 609 (100.0) |
| **Taking iron tablets, n (%)** | | |
| | Yes | 570 (93.6) |
| | No | 39 (6.4) |
| **Literate, n (%)** | | |
| | Yes | 557 (91.5) |
| | No | 52 (8.5) |
| **Literate husband, n (%)** | | |
| | Yes | 586 (96.2) |
| | No | 23 (3.8) |
| **Education level, n (%)** | | |

| | | |
|---|---|---|
| | Non-educated | 10 (1.6) |
| | Primary | 64 (10.5) |
| | Secondary | 215 (35.3) |
| | Higher secondary | 197 (32.3) |
| | Bachelor | 110 (18.1) |
| | Higher than Bachelor | 13 (2.1) |
| **Husband's education level, n (%)** | | |
| | Non-educated | 8 (1.3) |
| | Primary | 63 (10.3) |
| | Secondary | 231 (37.9) |
| | Higher secondary | 206 (33.8) |
| | Bachelor | 92 (15.1) |
| | Higher than Bachelor | 9 (1.5) |
| **Occupation, n (%)** | | |
| | Government job | 11 (1.8) |
| | Agriculture | 12 (2.0) |
| | Labor | 5 (0.8) |
| | Business | 36 (5.9) |
| | Housewife | 502 (82.4) |
| | Others | 43 (7.1) |
| **Husband's occupation, n (%)** | | |
| | Government job | 25 (4.1) |
| | Agriculture | 16 (2.6) |
| | Labor | 51 (8.4) |
| | Business | 126 (20.7) |
| | Migrant workers | 263 (43.2) |
| | Unemployed | 19 (3.1) |
| | Others | 109 (17.9) |
| **Household income, n (%)** | | |
| | Below 9,999Rs | 26 (4.3) |
| | 10,000–19,999Rs | 99 (16.3) |
| | 20,000–29,999Rs | 145 (23.8) |
| | 30,000–39,999Rs | 112 (18.4) |

| | | |
|---|---|---|
| | 40,000–49,999Rs | 62 (10.2) |
| | 50,000–59,999Rs | 59 (9.7) |
| | 60,000–69,999Rs | 19 (3.1) |
| | 70,000–79,999Rs | 11 (1.8) |
| | 80,000–89,999Rs | 9 (1.5) |
| | 90,000–99,999Rs | 10 (1.6) |
| | 100,000Rs or more | 57 (9.4) |

[a]1 Nepalese Rupee = 0.008269 United States Dollars (27[th] June 2020)

SD, standard deviation

**Table 2. WHO classification of anemia based on blood hemoglobin level (Hb), body mass index, and health literacy (n=138)**

| **Baseline** | **Average±SD (Range)** | **Median (IQR)** | | | |
|---|---|---|---|---|---|
| **BMI** | 25.2±4.8 (18.0–47.3) | 24.8 (4.7) | | | |
| **Hb (g/dl)** | 11.0±1.2 (6.0–14.1) | 11.0 (1.4) | | | |
| **Health literacy total score** | 57.6±10.0 (32.0–70.0) | 57.0 (19.0) | | | |
| **Functional literacy score** | 17.9±6.6 (5.0–25.0) | 20.0 (11.5) | | | |
| **Communicative literacy score** | 22.1±2.9 (11.0–25.0) | 22.0 (5.0) | | | |
| **Critical literacy score** | 17.7±2.1 (8.0–20.0) | 18.0 (4.0) | | | |
| **Baseline** | | | **Endline** | | |
| **Hb level g/dl** | Diagnosis | n (%) | Hb level g/dl | Diagnosis | n (%) |
| **<7.0 g/dl** | Severe anemic | 0 (0.0) | <7.0 g/dl | Severe anemic | 0 (0.0) |
| **7.0–10.9 g/dl** | Anemic | 138 (100.0) | 7.0–10.9 g/dl | Anemic | 37 (26.8) |
| **≥11 g/dl** | Non-anemic | 0 (0.0) | ≥11 g/dl | Non-anemic | 101 (73.2) |

SD, standard deviation; IQR, interquartile range; BMI, body mass index

The results of the confirmatory factor analysis on the health literacy scale applied to the scale developer’s model showed reasonable goodness of fit ($\chi^2$=347.090(64), $p<.0001$), and Kaiser–Meyer–Olkin measure of sampling adequacy (KMO=0.903). The factor loadings of all items are 0.614–0.962 and all Cronbach’s alpha coefficients for the subscales were over 0.970.

The mean Hb level at the first visit (8–12 weeks’ gestation) was 11.0 ± 1.2 g/dl (6.0–14.1), and 66 (47.8%) patients with anemia had Hb levels below 11.0 g/dl.

Hb levels before and after the intervention were compared by intervention in the three groups. The Hb levels before and after the intervention were respectively 10.20±0.62 g/dl and 11.65±0.59 g/dl in the education group, 10.19±0.69 g/dl and 11.15±1.08 g/dl in the material distribution group, and 10.27±0.51 g/dl and 11.14±1.19 g/dl in the control group. The results of a pre- and post-intervention comparison of the three groups showed a statistically significant difference ($P<0.035$) in mean Hb levels after the intervention. There was no significant difference in Hb levels among the three groups before the intervention ($P=0.804$).

The changes in Hb levels were 1.40 ± 0.84 g/dl, 0.96 ± 1.21 g/dl, and 0.87 ± 1.11 g/dl in the education, material distribution, and control groups, respectively (Table 3). The comparison of the rate of change among the three groups showed a statistically significant difference ($P<0.035$), with the education group having a statistically significantly higher rate of change.

Comparing changes in health literacy scale (total and subscale scores) before and after the intervention, there was a statistically significant difference in total health literacy scores for all three

groups (P<0.001). The median (interquartile range) pre- and post-intervention total scores were respectively 61.0 (54.52–69.75) and 108.50 (94.0–118.0) in the education group, 56.0 (51.25–70.0) and 96.0 (77.25–117.5) in the distribution group, and 58.50 (55.0–70.0) and 98.5 (96.0–120.0) in the control group (Table 4).

**Table 3. Comparison of primary outcome variables at baseline and endline in the three groups (n=138)**

| | | **Education group (n=49)** | **Distribution group (n=44)** | **Control Group (n=45)** | **P[a]** |
|---|---|---|---|---|---|
| | | Mean±SD | Mean±SD | Mean±SD | |
| **Hb level g/dl** | Baseline | 10.20±0.62 | 10.19±0.69 | 10.27±0.51 | 0.804 |
| | Endline | 11.65±0.59 | 11.15±1.08 | 11.14±1.19 | <0.035* |
| | Change | 1.40±0.84 | 0.96±1.21 | 0.87±1.11 | <0.035* |

[a]Analysis of variance (ANOVA) P<0.05*, P<0.001**

SD, standard deviation

Statistical significance was set at p<0.05.

**Table 4. Change in health literacy scale (total score and median subscale score (interquartile range)) before and after intervention (n=138)**

| | | **Baseline** | **Endline** | **Z-score** | **P[a]** |
|---|---|---|---|---|---|
| **Health literacy total score** | Education Group (n=49) | 61.0 (54.52–69.75) | 08.5 (94.0–118.0) | 1128.000 | <0.001** |
| | Distribution Group (n=44) | 56.0 (51.25–70.0) | 96.0 (77.25–117.5) | 820.000 | <0.001** |
| | Control Group (n=45) | 58.5 (55.0–70.0) | 98.5 (96.0–120.0) | 990.000 | <0.001** |
| **Functional literacy score** | Education Group (n=49) | 25.0 (19.25–25.0) | 25.0 (20.0–25.0) | 36.000 | <0.012* |
| | Distribution Group (n=44) | 22.5 (18.25–25.0) | 21.0 (18.25–25.0) | 31.500 | 0.892 |
| | Control Group (n=45) | 20.5 (19.0–25.0) | 21.0 (19.0–25.0) | 76.500 | 0.127 |
| **Communicative** | Education | 20.5 (20.0– | 22.0 (20.0– | 75.000 | <0.004* |

| | | | | | |
|---|---|---|---|---|---|
| **literacy score** | Group (n=49) | 25.0) | 25.0) | | |
| | Distribution Group (n=44) | 20.5 (20.0–25.0) | 20.5 (20.0–25.0) | 10.500 | 0.527 |
| | Control Group (n=45) | 22.0 (20.0–25.0) | 21.5 (20.0–25.0) | 14.000 | 1.000 |
| **Critical literacy score** | Education Group (n=49) | 17.0 (16.0–20.0) | 17.0 (16.0–20.0) | 28.000 | ＜0.014* |
| | Distribution Group (n=44) | 16.5 (16.0–20.0) | 16.5 (16.0–20.0) | 0.000 | 0.317 |
| | Control Group (n=45) | 17.0 (16.0–20.0) | 16.5 (16.0–20.0) | 0.000 | 0.157 |

[a] Wilcoxon signed rank test P<0.05*, P<0.001**
Statistical significance was set at p<0.05.

Only the education group showed statistically significant differences in functional literacy, communicative literacy, and critical literacy subscale scores. The median (interquartile range) pre- and post-intervention scores for functional literacy were respectively 25.0 (19.25–25.0) and 25.0 (20.0–25.2) in the education group (P<0.012, a statistically significant difference), 22.5 (18.25–25.0) and 21.0 (18.25–25.0) in the distribution group (P=0.892), and 20.50 (19.0–25.0) and 21.0 (19.0–25.0) in the control group (P=0.127, a statistically insignificant difference). The median (interquartile range) communication literacy scores before and after the intervention were respectively 20.5 (20.0–25.0) and 22.0 (20.0–25.0) in the education group (P<0.004, a statistically significant difference), 20.5 (20.0–25.0) and 20.5 (20.0–25.0) in the distribution group (P=0.527), and 22.0 (20.0–25.0) and 21.5 (20.0–25.0) in the control group (P=1.000).

The median (interquartile range) critical literacy scores before and after the intervention were respectively 17.0 (16.0–20.0) and 17.5 (16.5–20.0) in the education group (P<0.014, a statistically

significant difference), 16.5 (16.0–20.0) and 16.5 (16.0–20.0) in the distribution group (P=0.317), and 17.0 (16.0–20.0) and 16.5 (16.0–20.0) in the control group (P=0.157).

# Discussion

Iron deficiency anemia among Nepalese women is a serious public health problem that has been addressed but has yet to be resolved. The average age of first childbirth of the women in this study was as low as 21.1 ± 3.2 years, and more than half were in their teens, suggesting that anemia had persisted since adolescence. The study showed that hemoglobin levels and health literacy can be improved by creating text-free material and continuing face-to-face individualized health education from early pregnancy. The health education group showed significantly improved hemoglobin levels compared to the handout and control groups.

One art-based reproductive health intervention study in India [24] used a health literacy improvement approach to strengthen women’s decision-making power regarding contraceptive knowledge, marital communication, family planning decision-making, and women’s reproductive rights through street theater and puppet shows. Lori et al [25] also conducted a study in Ghana aimed at improving health literacy in order to increase the ability to understand health messages and practice healthy behaviors. They used a "take action" strategy using demonstrations, role-plays, and puppet shows to help women understand and practice health messages. The results showed that

health literacy improved in the intervention group compared with that in the group that received general maternity care. In addition, one prospective cohort study in Ghana [26] examined the effects of storytelling, peer support, demonstrations, and teach-back on improving health literacy. Prenatal and postpartum women in Ghana who participated in the study showed improved understanding of health messages and improved health literacy. Therefore, health literacy interventions targeting women in developing countries are likely to contribute to improved reproductive health.

The literacy rate of the participants in this study was more than 90% for both couples, which is comparable with the literacy rate of youth aged 15–24 years in Nepal [27]. Furthermore, 98.3% of the participants had at least primary-level education, indicating that the participants were capable of understanding health-related information. However, communicative literacy and critical literacy scale scores were low, suggesting that low health literacy may be related to one of the factors contributing to health issues remaining unresolved and becoming more complex, despite the growing educational system among the younger generation in Nepal.

Information is a necessary grounding material for personal decision-making. Autonomous decision-making, including obtaining reliable information, selecting the appropriate information for one's own situation from a list of options, and making autonomous decisions, is the desired health behavior. However, Nepalese women's autonomy in household decision-making is reported to be low in all aspects of managing their own health, making major household purchases, purchasing

daily necessities, and visiting family and relatives [28].

In other words, if we can strengthen health literacy with effective methodologies to empower Nepalese women to take interest in their own health, seek health information on their own, discern and select information, communicate it to others, and adapt it to their own lives, they will significantly contribute to maintaining and improving the health of not only for themselves but also their families.

Interventions for the younger generation are particularly useful and health behaviors are more likely to be sustained; thus, it is hoped that interventions to improve health literacy will become widespread as preconception care [29]. This study found that art-based material and face-to-face tutoring improved the health literacy of Nepalese pregnant women, and that improved health literacy contributed to improved anemia among pregnant women. Health literacy was found to be an important factor in improving the nutritional status of Nepalese pregnant women.

Currently, the nutritional challenges of Nepalese pregnant women are becoming more complex. Comprehensive nutrition assessment using multiple indicators is needed to determine the extent and characteristics of nutritional disorders. This indicates an urgent need for nutrition assessment and review of existing nutritional support to all generations in developing countries. This study focused on health literacy as well as literacy and an intervention. The study revealed the importance of health literacy as a determinant of the nutritional status of anemic Nepalese pregnant women and showed

the effectiveness of the intervention. The study revealed that health literacy is an important factor in supporting nutrition improvement for Nepalese pregnant women even though literacy rates have increased. Furthermore, the study showed the importance of evidence-based knowledge dissemination using effective educational material. The study employed material designed using pictures and diagrams that are easy to understand visually for pregnant women and families with low health literacy.

## Limitations

In this study, only a self-reporting procedure was used to confirm iron medication behavior, which limited tracking. There is a need to implement a research procedure to count iron tablet sheets in the future to accurately confirm medication adherence. In addition, this was a single-center cross-sectional survey study, and future long-term multicenter observational studies are desirable. Furthermore, to determine the cause of anemia, not only the hemoglobin level but also a comprehensive evaluation of other parameters, including hematocrit, mean corpuscular volume, red blood cell, mean corpuscular hemoglobin, mean corpuscular hemoglobin concentration, red cell distribution width, and ferritin, is necessary.

## Conclusions

The results of this study showed that individualized health education using pictures, photographs, and nomograms without text was effective in improving hemoglobin levels among pregnant women. A Nepali version of the HLS-14 was developed as a literacy measurement tool and found to be useful for evaluating health literacy levels before and after intervention. The women who received education sessions showed significantly higher improvement in overall health literacy, functional literacy, communicative literacy, and critical literacy scores than the distribution and control groups.

# Acknowledgments

We would like to express our deepest gratitude to the Nepalese pregnant women who participated in the survey. We also extend our utmost respect and appreciation for the active participation of the intervention study participants and the cooperation and support of the five research nurses.

This work was supported by JSPS KAKENHI Grant Numbers 18k17592,22k0026.

# References

1. Health Literacy: the Solid Facts. Health Literacy: the Solid Facts. Available from: who.int [Accessed 2022-11-10] ISBN: 978 92 890 00154.
2. People's Health Movement Global Secretariat. People's charter for health; 2018. [Cited 2022-11-10]. Available from: https://phmovement.org/the-peoples-charter-for-health/.
3. Hinson L, Edmeades J, Murithi L, Puri M. Developing and testing measures of reproductive decision-making agency in Nepal. SSM Popul Health. 2019;9: 100473. doi: 10.1016/j.ssmph.2019.100473.
4. Nutbeam D. The evolving concept of health literacy. Soc Sci Med. 2008;67: 2072-2078. doi: 10.1016/j.socscimed.2008.09.050.
5. Budhathoki SS, Pokharel PK, Good S, Limbu S, Bhattachan M, Osborne RH. The potential of

health literacy to address the health related UN sustainable development goal 3 (SDG3) in Nepal: a rapid review. BMC Health Serv Res. 2017;17: 237. doi: 10.1186/s12913-017-2183-6.

6. Coulter A. Patient engagement—what works? J Ambul Care Manage. 2012;35: 80-89. doi: 10.1097/JAC.0b013e318249e0fd.
7. Vahdat S, Hamzehgardeshi L, Hessam S, Hamzehgardeshi Z. Patient involvement in health care decision making: a review. Iran Red Crescent Med J. 2014;16: e12454. doi: 10.5812/ircmj.12454.
8. Stacey D, Bennett CL, Barry MJ, Col NF, Eden KB, Holmes-Rovner M, et al. Decision aids for people facing health treatment or screening decisions. Cochrane Database Syst Rev. 2011;10: CD001431.
9. Shrestha M, Shrestha L, Basnet S, Shrestha PS. Trends in perinatal mortality in Tribhuvan University Teaching Hospital: 13 years review. J Nepal Paediatr Soc. 2012;32: 150-153. doi: 10.3126/jnps.v32i2.6898.
10. Black RE, Victora CG, Walker SP, Bhutta ZA, Christian P, de Onis M, et al. Maternal and child undernutrition and overweight in low-income and middle-income countries. Lancet. 2013;382: 427-451. doi: 10.1016/S0140-6736(13)60937-X.
11. Levy A, Fraser D, Katz M, Mazor M, Sheiner E. Maternal anemia during pregnancy is an independent risk factor for low birthweight and preterm delivery. Eur J Obstet Gynecol Reprod Biol. 2005;122: 182-186. doi: 10.1016/j.ejogrb.2005.02.015.
12. McLean E, Cogswell M, Egli I, Wojdyla D, de Benoist B. Worldwide prevalence of anaemia, WHO vitamin and Mineral Nutrition Information System, 1993-2005. Public Health Nutr. 2009;12: 444-454. doi: 10.1017/S1368980008002401.
13. Rahman MM, Abe SK, Rahman MS, Kanda M, Narita S, Bilano V, et al. Maternal anemia and risk of adverse birth and health outcomes in low- and middle-income countries: systematic review and meta-analysis. Am J Clin Nutr. 2016;103: 495-504. doi: 10.3945/ajcn.115.107896.
14. Ministry of Health. Nepal; new ERA; and ICF. Nepal demographic and health survey 2016; 2017. Available from: www.dhsprogram.com/pubs/pdf/fr336/fr336.pdf. [Accessed 2022-11-10]. Kathmandu, Nepal: Ministry of Health, Nepal.
15. Ministry of Health. Nepal; New ERA, & ICF. Nepal Demographic and Health Survey; 2017. Available from: healthynewbornnetwork.org Kathmandu. Nepal: Ministry of Health, Nepal. (Accessed 20220-11-10).
16. Christian P, Shrestha J, Leclerq SC, Khatry SK, Jiang T, Wagner T, et al. Supplementation with micronutrients in addition to iron and folic acid does not further improve the hematologic status of pregnant women in rural Nepal. J Nutr. 2003;133: 3492-3498. doi: 10.1093/jn/133.11.3492.
17. Lamichhane R, Zhao Y, Paudel S, Adewuyi EO. Factors associated with infant mortality in Nepal: a comparative analysis of Nepal demographic and health surveys (NDHS) 2006 and 2011.

BMC Public Health. 2017;17: 53. doi: 10.1186/s12889-016-3922-z.
18. Carbone ET, Zoellner JM. Nutrition and health literacy: a systematic review to inform nutrition research and practice. J Acad Nutr Diet. 2012;112: 254-265. doi: 10.1016/j.jada.2011.08.042.
19. Meherali S, Punjani NS, Mevawala A. Health literacy interventions to improve health outcomes in low- and middle-income countries. Health Lit Res Pract. 2020;4: e251-e266. doi: 10.3928/24748307-20201118-01.
20. Michielutte R, Bahnson J, Dignan MB, Schroeder EM. The use of illustrations and narrative text style to improve readability of a health education brochure. J Cancer Educ. 1992;7: 251-260. doi: 10.1080/08858199209528176.
21. Suka M, Odajima T, Kasai M, Igarashi A, Ishikawa H, Kusama M, et al. The 14-item health literacy scale for Japanese adults (HLS-14). Environ Health Prev Med. 2013;18: 407-415. doi: 10.1007/s12199-013-0340-z.
22. Batista MJ, Marques ACP, Silva Junior MF, Alencar GP, Sousa MDLR. Translation, cross-cultural adaptation and psychometric evaluation of Brazilian Portuguese version of the 14-item Health Literacy Scale. Cien Saude Colet. Portuguese version. 2020;25: 2847-2857. doi: 10.1590/1413-81232020257.22282018.
23. Pokharel RK, Maharjan MR, Mathema P, Harvey PW. Success in delivering interventions to reduce maternal anemia in Nepal: a case study of the intensification of maternal and neonatal Micronutrient Program. Washington, District of Columbia: A2Z Project, United States Agency for International Development (USAID); 2011.
24. León FR, Lundgren R, Sinai I, Sinha R, Jennings V. Increasing literate and illiterate women's met need for contraception via empowerment: a quasi-experiment in rural India. Reprod Health. 2014;11: 74. doi: 10.1186/1742-4755-11-74.
25. Lori J, Munro ML, Ofosu-Darkwah H, Chuey M, Boyd C, Adanu R. Improving health literacy through facilitated group focused antenatal care. Ann Glob Health. 2015;81: 78. doi: 10.1016/j.aogh.2015.02.684.
26. Lori JR, Ofosu-Darkwah H, Boyd CJ, Banerjee T, Adanu RMK. Improving health literacy through group antenatal care: a prospective cohort study. BMC Pregnancy Childbirth. 2017;17: 228. doi: 10.1186/s12884-017-1414-5.
27. The estimation data for section "Nepal population literacy" is based on the latest data published by UNESCO Institute for Statistics. Nepal population; 2022 live — Countrymeters. Available from: https://countrymeters.info/en/Nepal (Accessed 20220-11-10).
28. Acharya DR, Bell JS, Simkhada P, van Teijlingen ER, Regmi PR. Women's autonomy in household decision-making: a demographic study in Nepal. Reprod Health. 2010;7: 15. doi: 10.1186/1742-4755-7-15.
29. Bhandari PM, Thapa K, Dhakal S, Bhochhibhoya S, Deuja R, Acharya P, et al. Breast cancer

literacy among higher secondary students: results from a cross-sectional study in Western Nepal. BMC Cancer. 2016;16: 119. doi: 10.1186/s12885-016-2166-8.

Figure 1 Click here to access/download;Figure;Fig1.tif

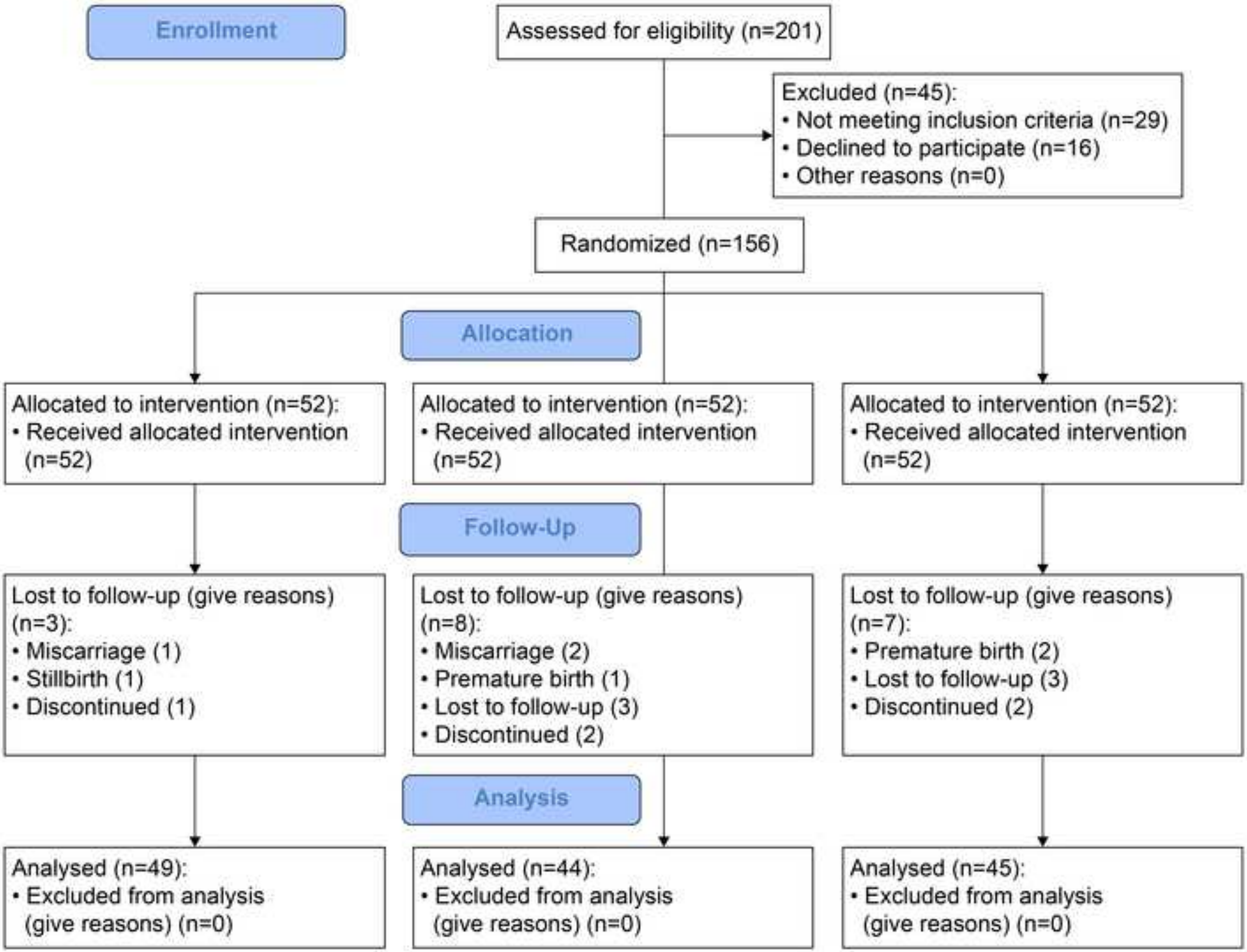

Click here to access/download
**Supporting Information**
CONSORT-2010-Checklist.doc

Click here to access/download
**Supporting Information**
Study Protocol.docx

Click here to access/download
**Supporting Information**
Ethics Committee Approval_Research Plan.pdf
