# Supplementary material for: Effectiveness of art-based health education on anemia and health literacy among pregnant women in Western Nepal: A randomized controlled trial
Source: PLoS One. 2024 Sep 30;19(9):e0281789. doi: 10.1371/journal.pone.0281789 (PMC11441646; doi:10.1371/journal.pone.0281789)
Supplement: Attachment — Submitted filename: Response_to_Reviewers.docx [file pone.0281789.s005.docx]

**Response to Reviewers**

We wish to express our appreciation to the Reviewer for the insightful comments, which have helped us significantly improve the paper.

1. Clinical trial registration appears to have been completed retroactively and should be clarified in Line105.

→Thank you for your suggestion. We have added the reasons for the delay in clinical trial enrollment in the revised manuscript (L121-124), as suggested by the Reviewer.

２. Ethics Committee approval is in both Japanese and English and it is difficult to review the CONSORT design (L120). Please provide additional copy in English.

→Thank you for providing these insights. We have provided the ethics approval document for this study in English.

３. In Data Collection/Sample Size, a previous study is mentioned (L276) and should be cited.

→We wish to express our appreciation to the reviewer for this insightful comment. We have cited the appropriate reference in the revised manuscript, according to the reviewer’s suggestion. The citation of Reference 13 in the previous version of the manuscript was a mistake, which we have now corrected by citing the appropriate reference (L294).

4. 　In Results and Table 1, the description of caste (L315-317) should match the description in Table 1.

→The authors would like to express their gratitude to the reviewers for their comments on this paper. We have revised the description of caste (L330-332) to match the description in Table 1.

5. In Table 1, “Occupation” (which addresses both husband and wife) could be moved next to “Occupation” for clarity

→We appreciate your suggestion. Table 1 has been revised.

1) your reasons for your delay in registering this study (after enrolment of participants started);

→We appreciate the reviewer’s comment on this point. The reasons for the delay in clinical trial enrollment have been included in the revised manuscript (L121-124).

2) confirmation that all related trials are registered by stating: “The authors confirm that all ongoing and related trials for this drug/intervention are registered”.

→Thank you for providing these insights. We confirm that the clinical trial is fully enrolled.

4. We noted in your submission details that a portion of your manuscript may have been presented or published elsewhere. [Yes, we are planning to write a report for the official publication of the Health Education Association of Nepal (HEAN) using Table 2.] Please clarify whether this [conference proceeding or publication] was peer-reviewed and formally published. If this work was previously peer-reviewed and published, in the cover letter please provide the reason that this work does not constitute dual publication and should be included in the current manuscript.

→ We thank the reviewers for their comments. We have not yet submitted a paper to HEAN. Following the publication of this paper, we intend to submit a paper to HEAN containing data from the participants in this study; however, the manuscript to be submitted to HEAN is for an entirely different purpose and will not duplicate any content in this manuscript. When submitting the paper to HEAN, we will cite this paper (if published) and explain the bibliographic information of this paper and the novelty of the newly submitted paper in a letter of commitment to be submitted to HEAN.

5. Thank you for stating the following in the Acknowledgments Section of your manuscript:

“This work was supported by JSPS KAKENHI Grant Numbers 18k17592,22k0026.”

We note that you have provided additional information within the Acknowledgements Section that is not currently declared in your Funding Statement. Please note that funding information should not appear in the Acknowledgments section or other areas of your manuscript. We will only publish funding information present in the Funding Statement section of the online submission form.

Please remove any funding-related text from the manuscript and let us know how you would like to update your Funding Statement. Currently, your Funding Statement reads as follows:

“• Initials of the authors who received each award: H S

• Grant numbers awarded to each author: Hiroko Sakai JSPS KAKENHI Grant Numbers 18k17592,22k0026.

• The full name of each funder: Grant-in-Aid for Young Scientists, Fostering Joint International Research (B)

• URL of each funder website: Japan Society for the Promotion of Science (jsps.go.jp)

The funders had no role in study design, data collection and analysis, decision to publish, or preparation of the manuscript.”

→We appreciate the reviewer’s comment on this point. According to the reviewer’s suggestion, the information on research grants has been removed from the Acknowledgements section. We also confirm that the grant information currently stated in the [Funding Statement] section is correct.

Please include your amended statements within your cover letter; we will change the online submission form on your behalf.

→We appreciate the reviewer’s comment on this point. There are no amendments or updates to the Funding Statement.

6. Please review your reference list to ensure that it is complete and correct. If you have cited papers that have been retracted, please include the rationale for doing so in the manuscript text, or remove these references and replace them with relevant current references. Any changes to the reference list should be mentioned in the rebuttal letter that accompanies your revised manuscript. If you need to cite a retracted article, indicate the article’s retracted status in the References list and also include a citation and full reference for the retraction notice.

→We have revised the manuscript according to the Reviewers’ suggestions. A mistake in reference 13 in the previous version of our manuscript has been corrected, and several new citations have been included in the revised manuscript.

**Response to Editor**

We wish to express our appreciation to the Editor for the insightful comments, which have helped us significantly improve the paper.

Additional Editor Comments (if provided):

This research regarding health education on anemia and health literacy among pregnant women in Nepal has merit but needs minor clarifications, i.e.,

Clinical Trial registration appears to have been completed retroactively, and should be clarified in Line 105 (L1050.

→We appreciate the editor’s comment on this point. We have clarified this in the revised manuscript. The reasons for the delay in clinical trial enrollment have been included in the revised manuscript (L121-124).

2. Ethics Committee approval is in both Japanese and English, and it is difficult to review the CONSORT design (L120).

→We are grateful for this point of view. We have submitted the English version of the Ethics Committee approval along with the revised manuscript.

3. In Data Collection/Sample Size, a previous study is mentioned (L276) and needs to be cited.

→We appreciate the reviewer’s comment on this point. The cited paper has been corrected, and the correct citation has been added to the revised manuscript. The citation of Reference 13 in the previous version of the manuscript was a mistake, which we have now corrected by citing the appropriate reference (L294).

4. In Results and Table 1, the description of caste (L315-317) should match the description in Table 1(L322).

→We thank the editor for the comments. We have revised Table 1 and the Results section so that the notation of the results is consistent (L330-332).

5. In Table 1, “Occupation” (which addresses both husband and wife) could be moved next to “Income” to prevent confusion.

→We wish to thank the editor for this comment. The notation has been appropriately corrected (Table 1) according to your suggestion.

**Response to Reviewers**

We appreciate your comments and insights related to the paper. We found your feedback very constructive. Our point by point responses to your comments are given below.

Reviewer #1: A three-arm randomized controlled clinical trial was conducted which aimed to assess the effectiveness of a face-to-face educational intervention in reducing anemia in pregnant Nepalese women. When comparing mean change in Hb levels from pre to post intervention the education group exhibited statistically significantly higher levels.

Minor revisions:

1- Abstract: Typographic error: Remove the phrase “in the group” which follows “0.96 +/- 1.21.”

→ We thank the reviewer for pointing this out. We have deleted this from the revised manuscript.

2- Line 290: For clarity consider stating, “Continuous variables were summarized using mean and standard deviation (SD).”

→We thank you for your comments in this regard. We have described this text according to the reviewer’s suggestion (L305-306).

3- Line 292: Grammatical error: Replace “between” with “among.”

→ We thank you for pointing this grammatical error out. We have revised this according to the reviewer’s suggestion (L307).

4- Line 297: To conform to convention, replace “probability values” with “p-values.”

→ We thank the reviewer for this comment. We have revised this according to the reviewer’s suggestion (L311).

5- Line 145 states that the results were analyzed using Microsoft Excel, and line 299 states that data were analyzed using SPSS. Please clarify.

→Thank you for pointing this out. We have revised this according to the reviewer’s suggestion (L161).

6- The standard statistical term for “average” is “mean.”

→Thank you for pointing this out to us. We have revised the term “average” to “mean” throughout the revised manuscript.

6- The p-value of 0.035 from the ANOVA test indicates statistically different means among the

three groups. Indicate the statistical method used for making pairwise comparisons to determine which pairs differed.

→We agree with you and have incorporated this suggestion throughout our paper.

We have re-analyzed the data in accordance with the reviewer’s suggestions.

The results of the new analysis have been added to Table 2 and the Results section (L352- 354) of the revised manuscript.

8- Table 4 results: State the statistical method and provide results for analyzing these four outcomes when comparing the three randomization arms.

→We have re-analyzed the data in accordance with the reviewer’s suggestions.

The results of the new analysis have been added to Table 3 and the Results section (L370-389) of the revised manuscript.

Reviewer #2: This finding add significant information to the literature of anemia prevention interventions Some improvements is needed in the introductions, method, results and discussions, please find the detail comment in the file provided

**Response to Reviewer 2**

Thank you very much for providing important insights. We are delighted to hear that you think our work will spark debate in our field. In the following sections, you will find our responses to your points and suggestions. We are grateful for the time and energy you have spent on our behalf.

１. Please add a prove (previous study) showing that health literacy showing that health literacy level affect maternal anemia(L85).

→We thank you for your important remark. We have added this information using previous studies that have shown an association between health literacy and pregnant women (L88-96).

2. How prevalence of low health literacy in Nepalese people? or Nepalese women in particular? or pregnant Nepalese women? please add date proved that statement.

this information is needed to add the urgency of health literacy intervention to prevent anemia in pregnancy(L86).

→ We used previous studies to add information on the health literacy of the Nepali people (L96-103).

3. Please add the number of clinical trial registration, after it is completely reviewed(L109).

→We appreciate the suggestion. The clinical trial registration number has been added (L123).

4. Please add information about who were blinded during the study? It is the participants or the investigator? (L122)

→Thank you for your important suggestions. We indicated in 159-160 who were blinded.

5. How frequent of this health education session?

Is it scheduled repeated sessions? or only one off meeting? How long the intervention period? (L174)

→We thank you for your important remarks. We have provided details of the health education provided during the three check-ups (L186-190).

6. to delivered this education content how long od each sessions given? and how much the frequency in total during the intervention period? (L193)

→Your comments are gratefully acknowledged. We have indicated the session times in L190.

7. Please add information,initially,how much respondent per group?

→We are grateful for your valuable suggestion. We have provided the number of participants in each of the initial three groups (L321-323).

8. To see the result of randomization, which was the cooperabilities of the characteristics among intervention and control group, please make cross tab the characteristics and between group(intervention dan controlled group) please add statistical value of comparability between variable of characteristics. (L322)

→We are grateful for your important suggestion. We had only presented the total information on study participants in the paper. We have improved Table 1 by presenting data for all three groups as suggested.

9. Please add the post hoc test (after ANOVA)for the endline data to add information about the significant data difference between three group.(L354)

→We appreciate your suggestion. The results of the Dunnett test have been added to L352-354, and Table 2 has also been revised.

10. Please add value of statistical test the different of health literacy score at base line. Please add　different of total health literacy score test at endline.(L 360)

→We gratefully acknowledge the suggestion. I have added the results of the statistical analysis to L370-373 and also revised Table 3.

11. What the implication of this finding to the implementation of delivery ANC service in the study area or policy of the delivery ANC service. (L420)

→We are grateful for your suggestions. I have added a discussion on the delivery of ANC service based on the findings of this study to L438-445.
