# Supplementary material for: Effectiveness of art-based health education on anemia and health literacy among pregnant women in Western Nepal: A randomized controlled trial
Source: PLoS One. 2024 Sep 30;19(9):e0281789. doi: 10.1371/journal.pone.0281789 (PMC11441646; doi:10.1371/journal.pone.0281789)
Supplement: Attachment — Submitted filename: Response to Reviewers.docx [file pone.0281789.s006.docx]

**Response to Reviewers**

Journal Requirements: Please review your reference list to ensure that it is complete and correct. If you have cited papers that have been retracted, please include the rationale for doing so in the manuscript text, or remove these references and replace them with relevant current references. Any changes to the reference list should be mentioned in the rebuttal letter that accompanies your revised manuscript. If you need to cite a retracted article, indicate the article’s retracted status in the References list and also include a citation and full reference for the retraction notice.

- We have checked the reference list and ensured that it is complete and correct. We confirm that no retracted papers have been cited.
